# 1455. Cluster of Methicillin-Susceptible *Staphylococcus aureus* (MSSA) Infections at an Outpatient Surgery Center

**DOI:** 10.1093/ofid/ofad500.1292

**Published:** 2023-11-27

**Authors:** Allison Bailey, Idamae Kennedy, Henning Ansorg, Noemi C Doohan, Shellie Robles-Davis, Juliet Stoltey, Erin Epson, Jane D Siegel

**Affiliations:** California Department of Public Health, San Francisco, California; California Department of Public Health, San Francisco, California; Santa Barbara County Public Health Department, Santa Barbara, California; Santa Barbara County, Santa Barbara, California; Santa Barbara County Public Health, Santa Barbara, California; California Department of Public Health, San Francisco, California; California Department of Public Health, San Francisco, California; California Department of Public Health, San Francisco, California

## Abstract

**Background:**

In February 2023, a local health department (LHD) notified the California Department of Public Health (CDPH) Healthcare-Associated Infections (HAI) Program of 3 patients hospitalized for methicillin-susceptible *Staphylococcus aureus* (MSSA) bacteremia and/or abscess following sacroiliac (SI) joint injections for pain at an outpatient surgery center. Two recovered after treatment and one expired 9 days after the injection. CDPH-HAI and the LHD conducted an investigation.

**Methods:**

LHD and CDPH infection preventionist staff went on site to evaluate infection control (IC) practices. Whole genome sequencing (WGS) was performed on all available MSSA isolates at the CDPH Microbial Diseases Laboratory.

**Results:**

Initial investigation revealed single-dose vials of one product had been used for multiple patients; this practice was discontinued. WGS found the 3 patient isolates were identical (0 single nucleotide polymorphism (SNP) difference). The same healthcare personnel (HCP) team had performed the procedures for all 3 infected patients. A site visit identified gaps in IC and injection safety practices. One HCP who provided care to all 3 patients had visible skin lesions on their hands, wore bracelets, and was unaware of a facility policy to report if they were unable to perform adequate hand hygiene. MSSA screening cultures were collected from 3 involved HCP and were positive in only the HCP with skin lesions. WGS showed that the HCP MSSA isolates were highly related to the patient isolates (0-2 SNP difference). We advised the facility to develop an IC program tailored to the procedures performed, including adverse event surveillance, adherence monitoring of hand hygiene and environmental services practices, and attention to injection safety. The MSSA colonized HCP was referred for treatment of their skin condition and decolonization and was restricted from patient care until their skin condition improved.

Phylogenetic tree for MSSA

**Figure:**
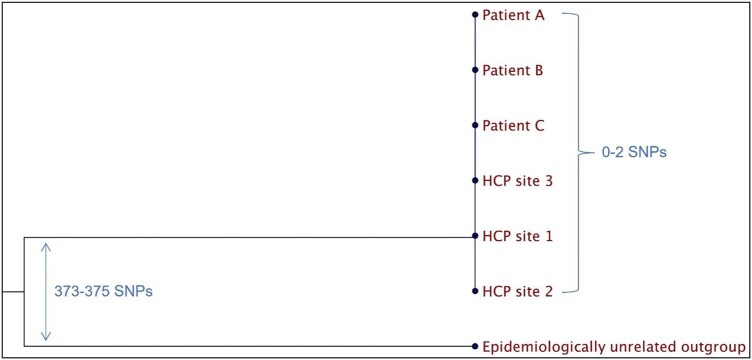

**Conclusion:**

A combination of injection safety and hand hygiene lapses led to MSSA transmission from an HCP to 3 patients. This cluster underscored the importance of IC programs in outpatient clinics and the risk of transmission of infectious agents from HCP to patients with gaps in IC practices. It also highlighted the contribution of WGS in investigating HAI outbreaks.

**Disclosures:**

**All Authors**: No reported disclosures

